# Supplementary material for: Observation of a singular Weyl point surrounded by charged nodal walls in PtGa
Source: Nat Commun. 2021 Jun 28;12:3994. doi: 10.1038/s41467-021-24289-0 (PMC8239007; doi:10.1038/s41467-021-24289-0)
Supplement: Supplementary file 1 — Supplementary Information [file 41467_2021_24289_MOESM1_ESM.pdf]

Supplementary Materials for

Observation of a singular Weyl point surrounded by charged Weyl nodal walls in PtGa

J.-Z. Ma[1,2*†], Q.-S. Wu[3,4*], M. Song[5,6], S.-N. Zhang[3,4], E. B. Guedes[1], S. A. Ekahana[1], M. Krivenkov[7], M. Y. Yao[8], S.-Y. Gao[9], W.-H. Fan[9], T. Qian[9], H. Ding[9,10], N. Plumb[1], M. Radovic[1], J. H. Dil[1,3], Y.-M. Xiong[5], K. Manna[8,11], C. Felser[8], O. V. Yazyev[3,4†], M. Shi[1†]

†Corresponding to: junzhama@cityu.edu.hk, oleg.yazyev@epfl.ch, ming.shi@psi.ch

This PDF file includes:

Supplementary Note 1.

Supplementary Table 1. Momentum space coordinates, energies and chirality of Weyl points in PtGa.

Supplementary Table 2. The velocities of the three kinds of Weyl nodes along high symmetry directions derived from ARPES and calculation data.

Supplementary Fig. 1. Projected band calculation along (001) direction.

Supplementary Fig. 2. Spin polarization EDC curves recorded with linearly vertical polarized (LH) light source.

Supplementary Fig. 3. The larger view of Fig. 4d,e,g,h.

Supplementary Fig. 4. The larger view of Fig. 4i.

Supplementary Fig. 5. Band structure of ReSi (space group No. 198) along the high-symmetry lines calculated with SOC included.

Supplementary Fig. 6. Band structure of high-pressure phase semiconductor Ge (space group No. 96) along the high-symmetry lines calculated with SOC included.

Supplementary Fig. 7. Band structure of $MgAs_4$ (space group No. 92) along the high-symmetry lines calculated with SOC included.

Supplementary Fig. 8. Band structure of α-phase TeO_2 (space group No. 92) along the high-symmetry lines calculated with SOC included.

Supplementary Fig. 9. The corresponding BZs of SGs a, No. 198, b, No. 96 and c, No. 92.

Supplementary Note 1

The band structure of PtGa in the vicinity of the Fermi level includes the 4-fold Rarita-Schwinger-Weyl (RSW) degeneracy between bands N-1 and N+2. Moreover, the complex arrangement of the Weyl points (WPs) and the Weyl nodal wall (WNW) between bands N-3 and N-2 is ravelled by the other 36 singular WPs at generic locations in k space. In order to simplify the electronic structure, here we discuss the TB model for an artificial lattice with space group 19 introduced in Ref. [13]. This model possesses screw symmetries $\{S_{2i} \mid i = x, y, z\}$. The composite symmetry $T{*}S_{2i}$ protects the band degeneracy at $k_i = \pi$, because $(T{*}S_{2i})^2 = -1$ leads to Kramer degeneracy on the boundary. Thus, the nodal wall on all boundaries of the cubic Brillouin zone is protected. On the other hand, time reversal symmetry protects the degeneracy at the BZ centre. So that the simple picture that a single Weyl point surrounded by nodal wall can be realised in this artificial model. In this model, there are only 8 bands with 2 near Fermi level without any RSW points (see Fig. 2c in Ref. [13]). The ideal nodal wall on the boundaries and node on the BZ centre are clearly resolved in the figure. There are no extra Weyl points appear on the generic k points. The bands that form the node at the Γ point have linear band dispersion in the k_x-k_y plane and along the Γ−Z direction. The Chern number or topological charge of this node calculated from Berry curvature is -1. This means that one quanta of Berry field flow into the BZ centre. Thus, this Weyl point is a singular Weyl point. The projected band structure on the (001) surface along the high symmetry-lines in Fig. 3a of Ref. [13] shows that except the broad bulk bands, there is no surface band that connects the projection of the single Weyl point. Also, no well-defined surface Fermi arc originating from the surface BZ centre can be found.

The reason for the absence of the surface Fermi arc connecting the projection of the Weyl point is the following. The Weyl nodal wall encloses the entire bulk BZ, so its projection covers the entire surface BZ. No matter how we choose the area near the Weyl point, the projection of the Weyl nodal wall cannot be avoided. From this perspective, the Chern number on any 2D slice is ill-defined, and the presence of the Fermi arc is not guaranteed.

Bearing in mind the analysis of single Weyl point surrounded by the Weyl nodal wall in the simple tight-binding model of Ref. [13], we reinvestigate the complex case of PtGa. There are 37 Weyl points of which 25 have positive chiral charges and 12 have negative chiral charges that are surrounded by the Weyl nodal wall. Their positions are listed in Supplementary Table 1. The experimental and calculated velocities of the related Weyl points are listed in Supplementary Table 2. One can naively suggest that the 12 negative WPs pair with 12 positive WPs of 25 available to form 12 Fermi arcs in the surface spectrum. However, as discussed above, since there is no well-defined Chern number in the 2D slice (Fig. 1a,b) of the 3D BZ, no surface Fermi arc connecting the projection of WPs with opposite charges is necessary. We calculated the band structure projected on the (001) surface of PtGa as shown in Supplementary Fig. 1b,c. It clearly shows that sharp well-defined surface bands connect the RSW points. However, we cannot find such surface bands that connect the projection of the single Weyl point at the Γ point. Although there is a signature of surface bands near the single Weyl point, these states are significantly broadened due to hybridization with bulk states. A similar situation can be seen in the iso-energy surface in Supplementary Fig. 1d. No surface Fermi arc connecting the projection of the single Weyl point can be observed.

Supplementary Table 1. Momentum space coordinates, energies and chirality of Weyl points in PtGa.

k_x (2π/a)	k_y (2π/a)	k_z (2π/a)	E-E_F (eV)	Chirality
±0.45578	±0.11501	±0.08524	–1.045	1
±0.08524	±0.45578	±0.11501	–1.045	1
±0.11501	±0.08524	±0.45578	–1.045	1
±0.42562	0	±0.09666	–0.948	–1
0	±0.09666	±0.42562	–0.948	–1
±0.09666	±0.42562	0	–0.948	–1
0	0	0	–0.409	1

Supplementary Table 2. The velocities of the three kinds of Weyl nodes along high symmetry directions derived from ARPES and calculation data. From ARPES data, velocities can be derived with the equation $\hbar v = \partial E/\partial k$ with $eV\dot{A}$ as unit. WP1 and WP2 belong to anisotropic type II Weyl point, so the left and right moving branches have different velocities.

$\frac{\partial E}{\partial k}$ $(eV\dot{A})$	WP0 along ΓX	WP0 along ΓM	WP1 along k_x	WP2 along k_y
ARPES	2.2	2.7	R: 1.3<br>L: 0.2	R:1.2<br>L: 0.4
calculation	2.3	2.3	R: 1.4<br>L: 0.3	R:1.0<br>L:0.4

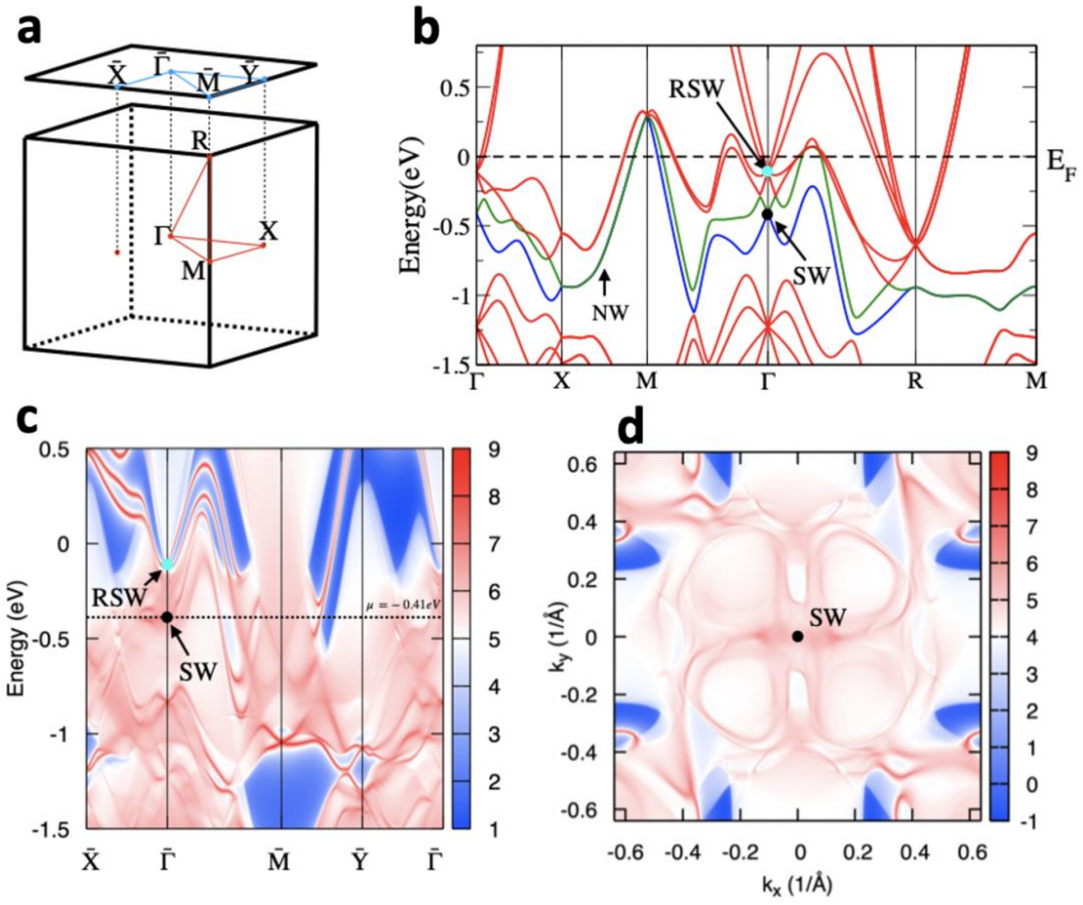

Supplementary Fig 1. Projected band calculation along (001) direction. a, Brillouin zone and projected Brillouin zone of the (001) surface of PtGa. **b**, Band structure of PtGa along a high-symmetry path. **c**, Band structure along high-symmetry path projected on the (001) surfaces of PtGa. The sharp surface bands connecting the RSW marked by the cyan dot in panels b and c are observed. However, no such bands can be observed for the single Weyl (SW) point marked by the black dot. **d**, Surface projected iso-energy spectrum of PtGa at chemical potential μ = –0.41 eV marked by the dashed line in panel c. No well-defined surface Fermi arc connecting the single Weyl point can be observed.

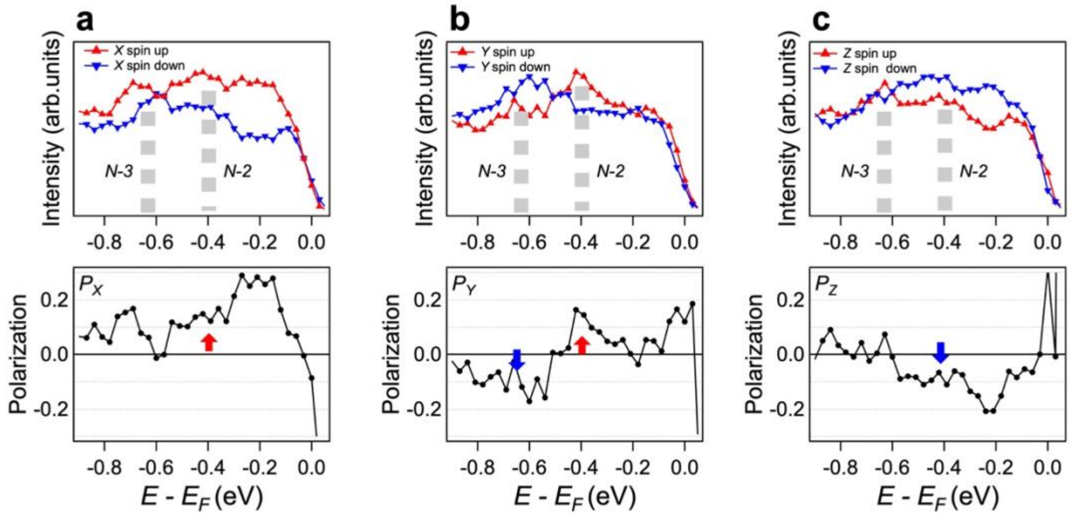

Supplementary Fig 2. Spin polarization EDC curves recorded with linearly vertical polarized (LH) light source. The *X*, *Y*, *Z* components of energy distribution curve (EDC) with the intensity of spin-up, spin-down, and spin polarization are shown along the EDC (green line in Fig. 3c). The spin polarization results are same as that recorded with CP light source in Fig. 3h,i,j, which means the spin polarization is not induced by the final state effect.

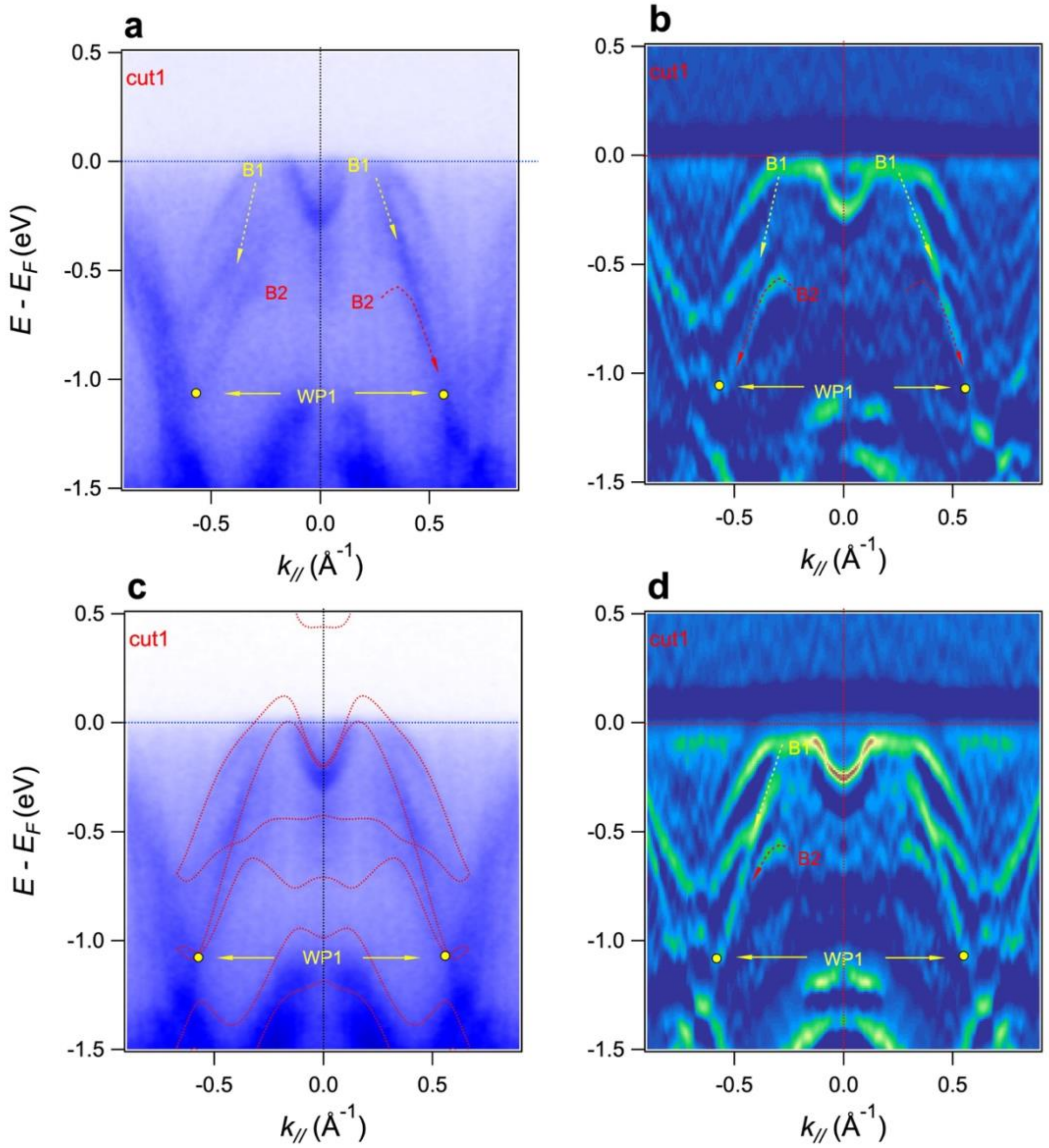

Supplementary Fig 3. The larger view of Fig. 4d,e,g,h. a and **b**, The raw ARPES spectra along cut1 and its curvature intensity plot, respectively. The data is identical to that plotted in Fig. 4g and h. **c** and **d**, The symmetrized ARPES spectrum of a, and its curvature intensity plot, respectively. The data is identical to in Figs. 4d and e. The bands calculated using LDA renormalized by a factor 1.2 and shifted up by 80 meV are overlaid in c for comparison.

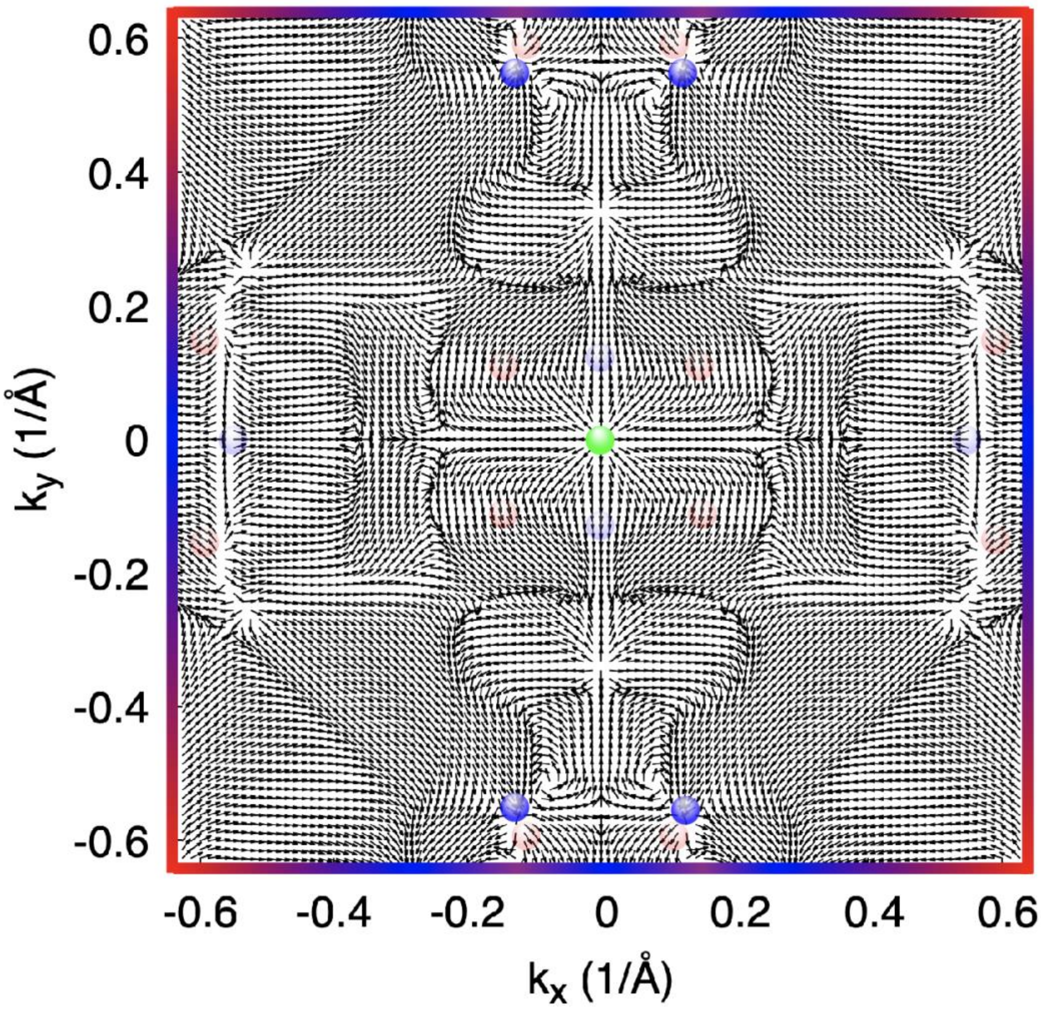

Supplementary Fig 4. The larger view of Fig. 4i. The in-plane component of the Berry curvature field in the $k_z = 0$ plane with the projections of the WPs overlaid are shown. The colors at the BZ boundary show the distribution of the topological charge on WNW.

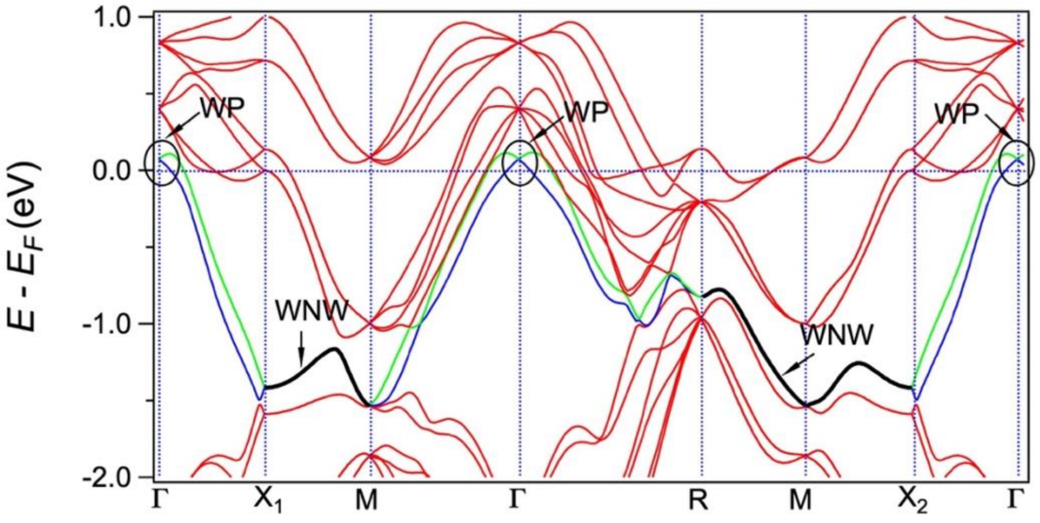

Supplementary Fig 5. Band structure of ReSi (space group No. 198) along the high-symmetry lines calculated with SOC included. The single unpaired Weyl point in the BZ center is very close to the Fermi level. This material is a promising candidate for transport measurements.

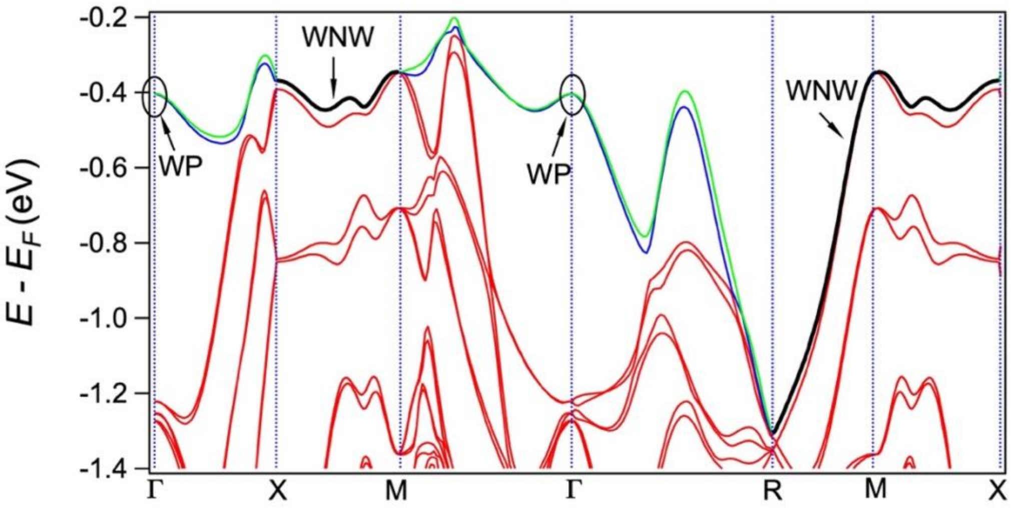

Supplementary Fig 6. Band structure of high-pressure phase semiconductor Ge (space group No. 96) along the high-symmetry lines calculated with SOC included. The band gap is 0.46 eV.

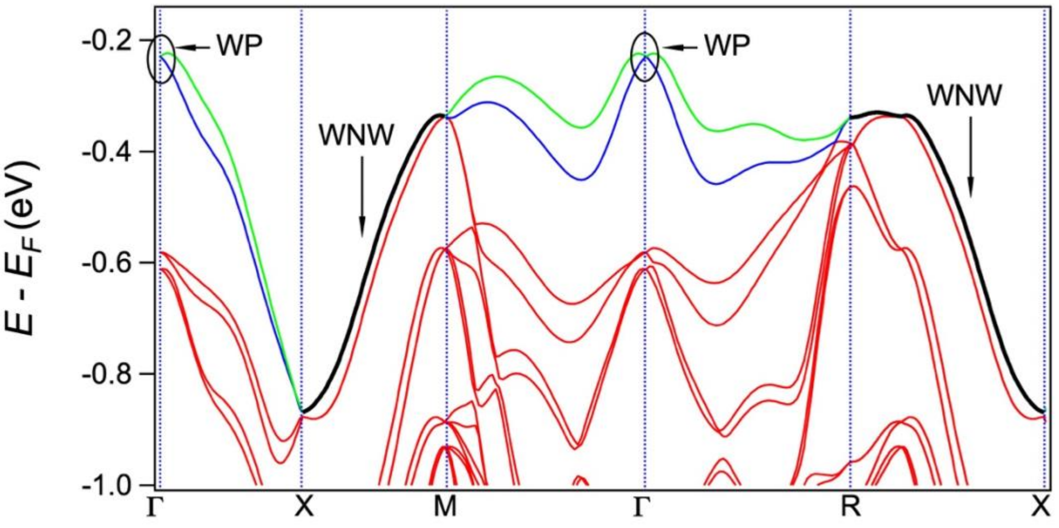

Supplementary Fig 7. Band structure of $MgAs_4$ (space group No. 92) along the high-symmetry lines calculated with SOC included. The band gap is 0.85 eV.

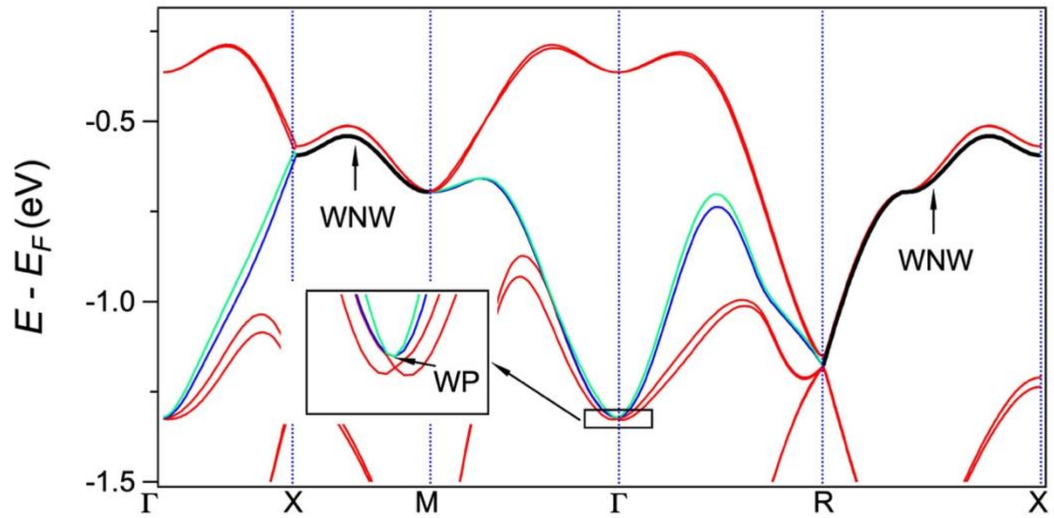

Supplementary Fig 8. Band structure of α-phase TeO_2 (space group No. 92) along the high-symmetry lines calculated with SOC included. The band gap is 2.41 eV.

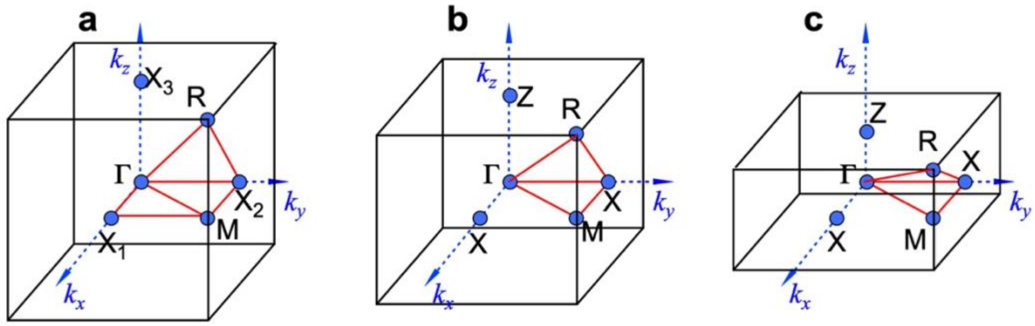

Supplementary Fig 9. The corresponding BZs of SGs **a**, No. 198, **b**, No. 96 and **c**, No. 92.
